# Dose-dependent roles of aspirin and other non-steroidal anti-inflammatory drugs in abnormal bone remodeling and skeletal regeneration

**DOI:** 10.1186/s13578-019-0369-9

**Published:** 2019-12-23

**Authors:** Yong Xie, Meng Pan, Yanpan Gao, Licheng Zhang, Wei Ge, Peifu Tang

**Affiliations:** 1 Department of Orthopedics, Chinese PLA General Hospital, Beijing, 100853 China; 2 State Key Laboratory of Medical Molecular Biology and Department of Immunology, Institute of Basic Medical Sciences, Chinese Academy of Medical Sciences, Beijing, 100005 China

**Keywords:** Aspirin, Bone remodeling, Osteoclast, Osteoblast, NSAIDs

## Abstract

The failure of remodeling process that constantly regenerates effete, aged bone is highly associated with bone nonunion and degenerative bone diseases. Numerous studies have demonstrated that aspirin and other non-steroidal anti-inflammatory drugs (NSAIDs) activate cytokines and mediators on osteoclasts, osteoblasts and their constituent progenitor cells located around the remodeling area. These cells contribute to a complex metabolic scenario, resulting in degradative or synthetic functions for bone mineral tissues. The spatiotemporal effects of aspirin and NSAIDs in the bone remodeling are controversial according the specific therapeutic doses used for different clinical conditions. Herein, we review in vitro, in vivo, and clinical studies on the dose-dependent roles of aspirin and NSAIDs in bone remodeling. Our results show that low-dose aspirin (< 100 μg/mL), which is widely recommended for prevention of thrombosis, is very likely to be benefit for maintaining bone mass and qualities by activation of osteoblastic bone formation and inhibition of osteoclast activities via cyclooxygenase-independent manner. While, the roles of high-dose aspirin (150–300 μg/mL) and other NSAIDs in bone self-regeneration and fracture-healing process are difficult to elucidate owing to their dual effects on osteoclast activity and bone formation of osteoblast. In conclusion, this study highlighted the potential clinical applications of low-dose aspirin in abnormal bone remodeling as well as the risks of high-dose aspirin and other NSAIDs for relieving pain and anti-inflammation in fractures and orthopedic operations.

## Background

Bone remodeling is a constant process that contributes to renewal of effete, aged bone and repair of the micro-damage of bone architectural integrity throughout life [1]. It couples the destructive process of bone resorption by teams of osteoclasts with bone synthesis by osteoblasts [2], and a period called reversal phase that separates bone-resorbing process from bone formation for several weeks [3]. Osteoclasts are derived from hematopoietic stem cells (HSCs), are unique for resorbing bone matrices, and share precursors with macrophages in the present knowledge [4]. In contrast, osteoblasts and reversal cells proven identical with osteoprogenitors (precursors of osteoblastic cells) [5] are of mesenchymal stem cell (MSC) origin [6]. The reversal cells are sort of mononucleated cells that colonize 80% of eroded bone surfaces undergoing remodeling [7]. It has been identified that the reversal cells are specific osteoblastic lineage cells, which play pivotal roles in coupling bone-resorption propitious to bone formation during a reversal phase [3, 8]. The organized functions of these cells is basic for keeping up physiological bone remodeling and advancing skeletal regeneration, which is strictly controlled by molecules or regulators such as the receptor activator of nuclear factor-κB ligand (RANKL), macrophage-colony stimulating factor [9], and activated T lymphocytes. Failure of bone remodeling in conditions with increased osteoclast activity or downregulated generation of the osteoblast lineage [10] leads to degenerative bone diseases such as osteoporosis, as well as increase the risk for delayed healing or nonunion of fractures. Previous epidemiological studies have demonstrated that taking of aspirin regularly is associated with changes in bone mineral density (BMD) and the fracture-healing processes. Further in vitro and in vivo investigations found that the aspirin participates in the regulation of bone remodeling when applied at the therapeutic doses.

Aspirin, also called acetylsalicylic acid, is known as a group of medications that belongs to nonsteroidal anti-inflammatory drugs (NSAIDs) and inhibits cyclooxygenase-1 (COX-1) and COX-2 enzymes in an irreversible manner [11]. COX-1 participates in physiological functions constitutively, whereas in pathophysiological processes such as pain, inflammation, and fever, COX-2 is inductively expressed and initial enzymatic activity converting arachidonic acid into prostaglandin E2 (PGE2) [12], which play pivotal roles in the promotion of pain and damage [13–15]. The high-dose aspirin (> 1000 mg per day) inhibits COX-2 more potently than COX-1 [16], and generally used for alleviation of pain and inflammatory response [17]. While low-dose aspirin (75–100 mg per day) inhibits COX-1 isozyme more strongly than COX-2 [11]. Such low doses could achieve persistent inhibition of platelet COX-1, preventing the formation of PGH2, and therefore thromboxane A2 (TXA2). It is widely recommended in prevention of acute coronary syndromes (ACS) and stroke in patients at high risk of developing blood clots [18, 19]. Low dose of aspirin is also considered to be alternative to other thrombosis phylactic agents following orthopedic operations [20]. The aspirin concentration in plasma usually ranges from 150 to 300 μg/mL in patients taking regular, high doses of aspirin and < 100 μg/mL after intake of therapeutic low doses [21].

Prior studies have shown that high-dose aspirin is related to the independent stimulation of osteoclast and osteoblast activity to destroy and generate bone tissues [22]. In addition, low-dose aspirin was shown to be associated with regulation of bone cells. Although its dose-dependent roles are conflicting and the detailed functional mechanisms of its regulation have not been fully elucidated, aspirin may exert multiple biological effects on bone remodeling [23]. In contrast to aspirin, other NSAIDs inhibit the COX enzymes reversibly [16], which leads to a modest increase in BMD of the hip and lumbar spine [23]. However, the conventional taking of NSAIDs as an analgesic seems to be negatively associated with union of long-bone fracture and the spinal-fusion rate [24]. Evidence of the relationship between NSAIDs use and bone remodeling is inconclusive. Here, we comprehensively reviewed the multiple roles of aspirin in bone remodeling and skeletal regeneration and the mechanism of actions by which aspirin may affect bone cells, especially in varying dose-dependent manners. In addition, we discuss the functions of different kinds of NSAIDs in bone remodeling and fracture healing, particularly their potential therapeutic effects or side effects during clinical applications in bone disorders.

### Low-dose aspirin may benefit for bone remodeling and skeletal regeneration

Mesenchymal stem cell-based intervention with low-dose aspirin (< 100 μg/mL) may benefit osteoporosis treatment by inhibition of osteoclast differentiation and activities [25]. Aspirin at a dose of 50 μg/mL can partially block the formation of osteoclasts induced by RANKL and led to a significantly decreased number of tartrate-resistant acid phosphatase (TRAP)-positive osteoclasts. When low-dose aspirin was continuously administered to ovariectomized (OVX) mice for 3 months, their femurs showed a higher level of BMD than the control group at 4 weeks of observation [26]. In addition, a markedly number decrease of the TRAP-positive cells in the trabecular bone of OVX mice and the serum levels of RANKL were detected [25]. Further examination of osteoclast activity showed that several serum markers were systemically changed by long-term taking aspirin at a low dosage, RANKL levels reduced, and osteoprotegerin (OPG) levels increased in OVX mice, which are known to be crucial for osteoclast differentiation [25]. In terms of stable ligand-activation of TXA2 receptors can induce osteoclast-like cell formation in murine bone marrow cultures [27], inhibition of TXA2 synthesis by low dose of aspirin might decrease osteoclastogenesis and bone resorption. These findings all indicated that low-dose aspirin can inhibit osteoclast differentiation and activity.

**Fig. 1:**
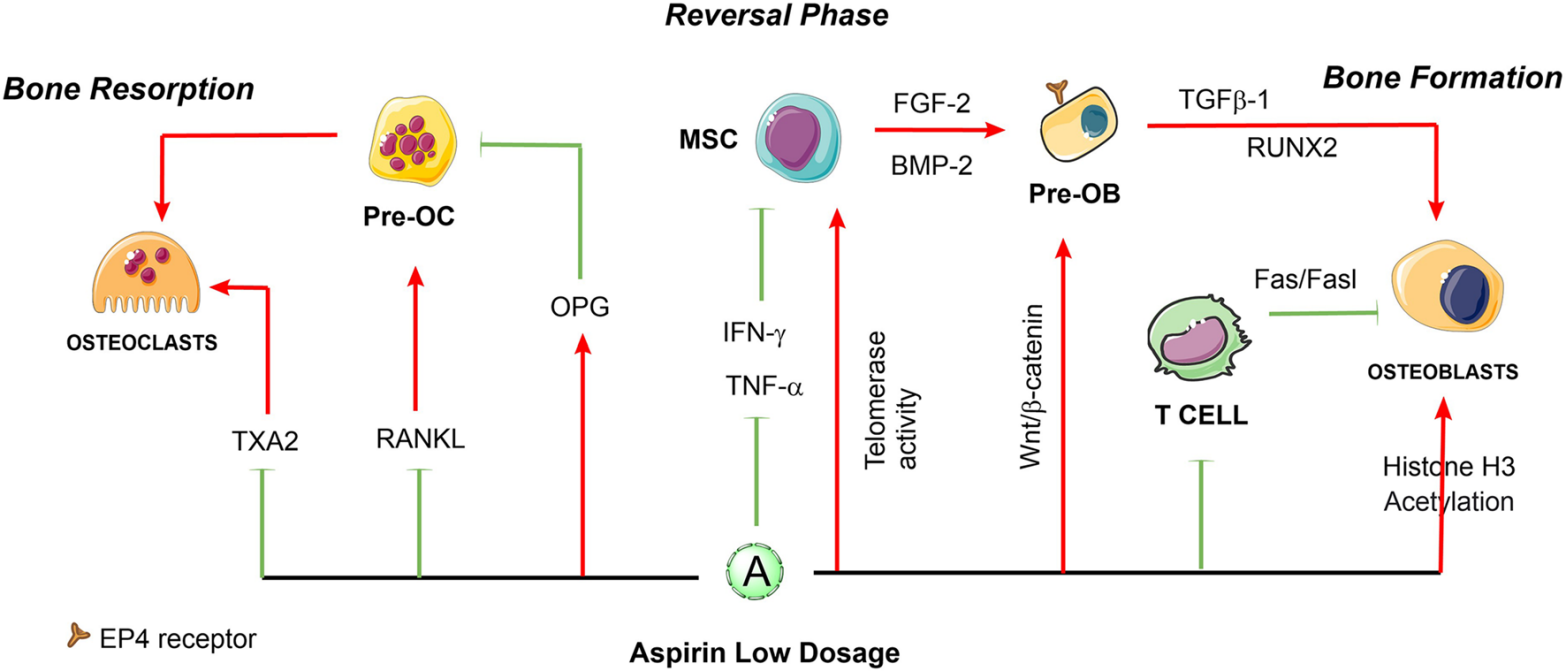
The roles of low-dose aspirin in the regulation of bone remodeling. Aspirin at low dosage might suppress the differentiation of osteoclasts and promotes the bone formation via osteoblastic cells. The solid red arrows indicate the promotion of cellular processes, and the solid green lines indicate inhibition of cellular processes. The dotted lines indicate that the mechanism has not been fully elucidated. *HSC* hematopoietic stem cells, *MSC* mesenchymal stem cells, *T cell* T lymphocytes, *Pre-OC* precursors of osteoclasts, *Pre-OB* precursors of osteoblasts, *OPG* osteoprotegerin

Additionally, low-dose aspirin was demonstrated to rescue impaired bone marrow MSC (BMMSCs) function by increasing the number of colony-forming unit fibroblasts and osteogenic capacities. Cultured BMMSCs treated with 50 μg/mL aspirin showed improvement of anti-apoptotic capacity and elevation of mineralized tissue formation in vitro and in vivo [26]. Aspirin at a dose of 50 μg/mL could also inhibit Fas/Fas ligand mediated apoptosis of BMMSCs via decrease viability of T-lymphocyte activation [25]. Moreover, in vitro low dose of aspirin significantly improved stem cell functions and prevented replicative senescence to improve bone formation [28]. Furthermore, aspirin at a dose of 75 μg/mL has been reported to enhance the immunomodulatory capacity of MSCs [29] and improve MSC-based tissue regeneration [30] by significantly reducing the expression of interferon-γ (IFN-γ) and tumor necrosis factor-α (TNF-α) [31]. The study elucidated that the use of low-dose aspirin slightly upregulates telomerase activity in BMMSCs and elongates their telomere lengths to avoid replicative senescence and improve stem cell functions [28], and has the capability to increase bone formation [28]. The underlying mechanisms of aspirin seem to have an association with increased expression of certain osteogenic genes such as *Runx2*, a master gene for osteogenic differentiation. Additionally, in vitro aspirin treatment accelerated degradation of phospho-β-catenin, leading to increased *Wnt* signaling, which is involved in osteogenesis [32]. In addition, aspirin at a low dose is likely to acetylate histones H3 in addition to the COX isoenzymes [33]. Acetylation of histones H3 and H4 is functionally coupled with chromatin-remodeling events that mediate the developmental induction of osteocalcin gene during osteoblast differentiation [34]. In contrast, the aspirin (< 100 μg/mL) could reverse the down-regulated histone deacetylases activity and induce inhibition of BMMSCs adipogenesis [35]. Moreover, low-dose aspirin exhibited excellent chemotactic effects in vitro [36]. The study of Tang et al. [37] demonstrated that both 50 μg/mL and 100 μg/mL aspirin significantly increased transforming growth factor β-1 (TGF-β1) production of human BMMSCs, then induces migration of MSCs to the bone remodeling sites [38]. In the latest studies of Sien et al., the OVX rats orally administered with low dose of aspirin (9 mg/kg/day, equivalent to 100 mg/day of human dose) showed less bone loss by using Micro-CT and histomorphometry. However, their in vitro results indicated that aspirin at low dose may increase the mineral component (calcium) of bone but be unfavorable for the synthesis of organic component (collagen), which result in a disorder in composition of bone, then exhibited no distinct tendency for improvement in bone mechanical properties [39]. These findings indicate that low-dose of aspirin can enhance the osteogenic capacities of MSCs and may rescue the bone loss from abnormal bone remodeling, while its mechanical properties need to be further detected. In general, Fig. 1 presents a schematic diagram of the major roles of low-dose aspirin in regulating the balance of bone remodeling to the direction of osteogenesis.

### Dual effects of high-dose aspirin on osteoclasts and osteoblasts activities

**Fig. 2:**
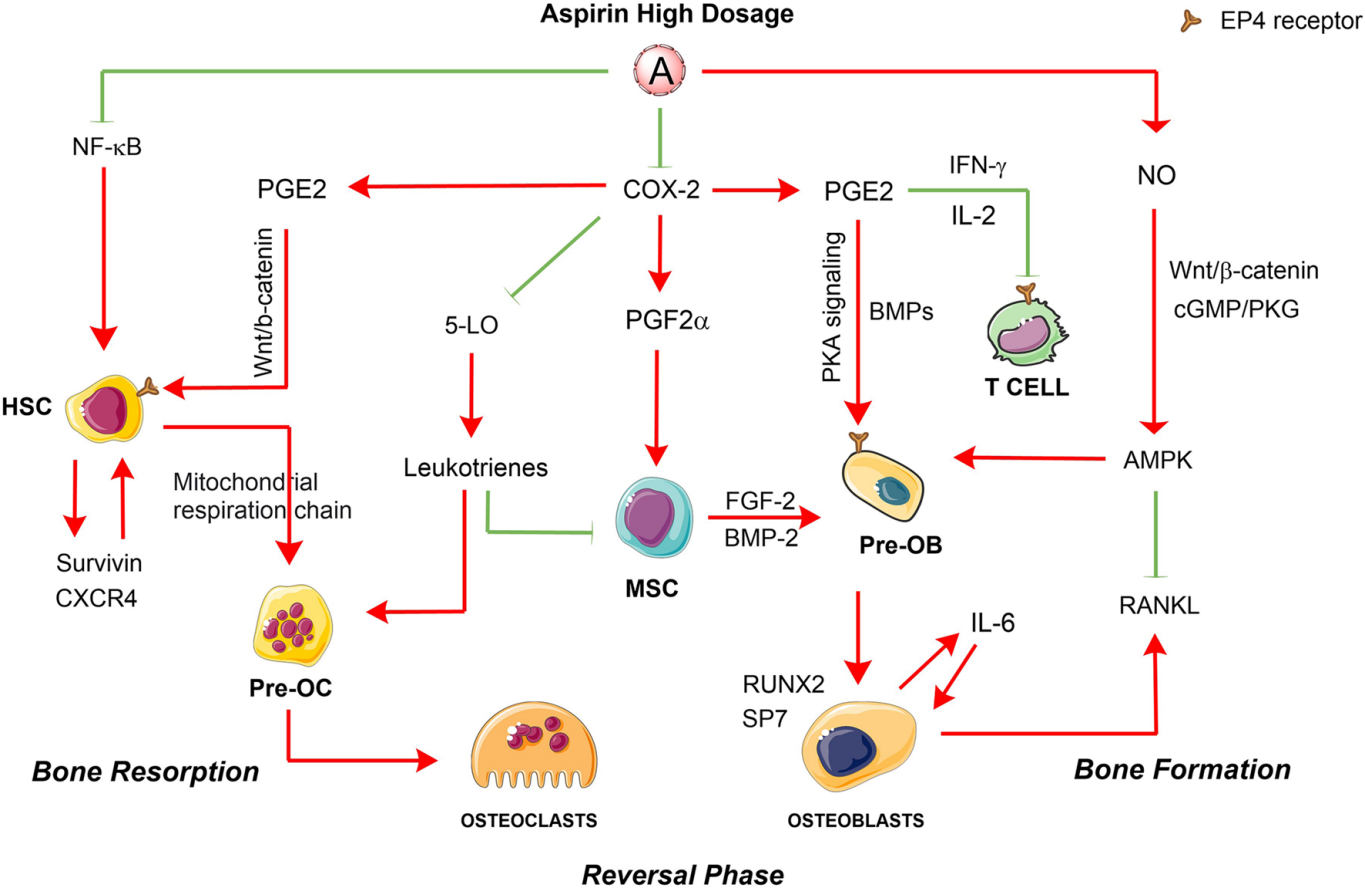
Dual effects of high-dose aspirin on osteoclasts and osteoblasts activities. Aspirin at high dosage regulates osteoclast-mediated bone resorption and osteoblastic bone formation by activating or inhibiting molecules and target cells. High-dose aspirin has multiple roles in the regulation of osteoclasts and osteoblasts. The solid red arrows indicate the promotion of cellular processes, and the solid green lines indicate inhibition of cellular processes. *HSC* hematopoietic stem cells, *MSC* mesenchymal stem cells, *T cell* T lymphocytes, *Pre-OC* precursors of osteoclasts, *Pre-OB* precursors of osteoblasts

In contrast to low-dose aspirin, high doses of aspirin acts COX-2-dependent inhibition, or through mechanisms such as formation of nitric oxide (NO) radicals [23], modulation of nuclear factor (NF)-κB, and electron transport chain pathways, which are involved in bone remodeling [40]. Consistent with these findings, in vitro and in vivo studies (Fig. 2) have confirmed that regular, high doses of aspirin have multiple effects on both osteoclasts and osteoblasts activities [41]. COX-2 is an essential player in both intramembranous and endochondral osteogenesis. The skeletal repair was significantly delayed in COX-2 knockout mice compared with COX-1-knockout and wildtype mice. When used at high doses for anti-inflammatory purposes, aspirin may have strong effects on bone remodeling, because of that the production of PGs is primarily mediated by COX-2 in osteoblasts [42]. PGs including PGE2, PGD2, and PGF2α belong to a group of lipid mediators that perform different functions in the regulation of homeostasis and inflammation. PGs act by activating the prostanoid receptor subfamily, which consists of eight members: the PGE receptors EP1, EP2, EP3, and EP4; the PGD receptor DP1; the PGI receptor (IP); the PGF receptor; and the thromboxane receptor [43]. PGs have been proved to active osteoblasts and osteoclasts directly in bone healing process [44]. In a rabbit ulnar osteotomy model, aspirin was demonstrated that delayed bone union with a threshold equivalent to a human dose of 325 mg [45].

PGE2 has direct, stable effects on long-term repopulating HSCs and promotes HSC implantation by up-regulating the chemokine receptor CXCR4 [46]. PGE2-induced Wnt/β-catenin signaling contributes to HSC development and regeneration [47]. Additionally, PGE2 enhances expression of survivin, an anti-apoptotic protein in the HSC niche [48]. With regard to the osteoblast/osteoclast differentiation and activities, studies have shown that PGE2 and PGF2α play a stimulating and inhibiting role in bone remodeling in an autocrine and/or paracrine manner [49, 50]. Aspirin at high concentration (> 200 μg/mL) has been demonstrated to have anti-proliferative effect on BMMSCs [37]. Chikazu et al. [51] demonstrated that COX-2 is involved in bone morphogenetic protein 2 (BMP-2)-induced osteoblast differentiation during ectopic bone formation. Correspondingly, Zhang et al. [52] discovered that bone marrow cell cultures from COX-2-knockout mice formed less osteoblasts than wild-type mice, while PGE2 and BMP-2 treatment could reverse this phenotype. Further study demonstrated that PGE2 may cooperate with BMPs to increase RUNX2 and SP7, two essential transcription factors required for bone formation [52]. Minamizaki et al. [53] indicated that the anabolic effect of PGE2 on osteogenesis are mediated partly by activation of specific EP4 receptors, which have been identified in human osteoblastic lineage [54]. In addition, the other studies suggested that selective EP4 receptor agonists could accelerate BMP-induced mineral nodule formation by stimulating the commitment and differentiation of osteoblast cell lines [55], within the involvement of protein kinase A (PKA) signaling pathway [56]. However, COX-2 deficient mice have satisfactory post-natal skeletal development, suggesting that the loss of COX-2 activity may not prevent stem cells from differentiating into osteoblasts. PGE2 can act as an immune suppressor of T-lymphocytes in a dose-dependent manner that involves EP4 receptors, and this immunosuppressive effect of PGE2 is correlated with IL-2 and IFN-γ inhibition [57]. Early in vitro studies have shown that PGF2α enhances the expression of IL-6 in osteoblasts [58]. Suda et al. have also reported that osteoblast formation was stimulated by IL-6 with induction of RANKL expression via a PGE2-related mechanism [59]. Previous studies have shown that PGF2α acts as a strong mitogenic and survival agent on osteoblasts, and these effects are, at least in part, mediated by fibroblast growth factor 2 (FGF-2), an anabolic bone agent that enhances bone formation by exerting an stimulation of the proliferation and differentiation of MSCs [60]. COX-2 inhibition transfers arachidonic acid to the 5-lipoxygenase (5-LO) pathway [61]. 5-LO is already known to be key factors in the enzymatic production of leukotrienes from arachidonic acid [62]. Leukotrienes can reduce the proliferation and activity of osteoblast in vitro while stimulating the formation and activity of osteoclast [63]. Consistent with the arachidonic acid-shunting mechanism, the levels of leukotriene B4 were found to be higher in fracture callus of COX-2 null mice than wide-type group. Together, inhibition of COX-2 activity by high-dose aspirin might decrease osteoblast differentiation or stimulate osteoclast activity [64, 65].

More recent data suggest that high-dose aspirin modulates signaling by suppressing NF-κB, a transcription factor complex, playing a pivotal role in many biological processes [66]. The NIK and RelB-dependent the receptor activator of nuclear factor-κB (RANK)-activated alternative NF-κB pathway controls osteoclast differentiation, mitochondrial biogenesis, and the respiratory chain [67], through independent pathways [68]. Oxidative phosphorylation in mitochondria seems to be the main bioenergetic source for osteoclast differentiation [69]. In addition, aspirin (oral dose of 200 mg/kg in mice) induces the formation of NO radicals, which independently reduces inflammation [70]. NO activates soluble guanylate cyclase to generate cGMP, which, in turn, stimulates production of protein kinase G (PKG). It also mediates the pro-survival effects of estrogens and mechanical stimulation in osteoblasts via cGMP/PKG signaling [71]. In murine primary osteoblasts, NO increases intracellular cGMP, Wnt/β-catenin signaling, and osteoblastic gene expression and protects cells from apoptosis [72]. NO-induced autophagy functions as a survival mechanism via adenosine monophosphate-activated protein kinase (AMPK) activation against apoptosis in the MC3T3-E1 (pre-osteoblast) cells [73]. A previous study showed that high-dose aspirin activates AMPK, a central regulator of cell growth and metabolism [74], which may inhibit the RANK/RANKL signaling, thereby explaining the mechanism of AMPK-mediated inhibition of osteoclastogenesis [75]. However, another study demonstrated that high-dose aspirin reduced osteoblast growth via cell cycle arrest and apoptosis induction, whereas low-dose aspirin had no effect on osteoblast growth [76]. Therefore, the underlying dose-dependent mechanism of aspirin might be responsible for this difference in its effects on bone cells [41].

### Differences and similarities between the roles of high-dose aspirin and other NSAIDs in in bone remodeling and fracture healing

The mechanism of action of other NSAIDs differs from that of high-dose aspirin [11]: NSAIDs inhibit the COX enzymes in a reversible manner, whereas high-dose aspirin inhibits the COX enzymes irreversibly [16]. NSAIDs are classified according to their mechanism of action or chemical structure, such as selective COX-2 inhibitors and acetic acid, propionic acid, and enolic acid (Oxicam) derivatives. These NSAIDs tend to have similar characteristics and tolerability within a group. Short-term use of therapeutic doses of NSAIDs regulated the differentiation activity of osteoblast-like cells and reduced the synthesis of basic phosphates and matrix mineralization [77]. Be similar to high-dose aspirin, NSAIDs affect osteoblasts by reducing the synthesis of PGs, resulting in inhibition of COX-2 enzymes [78]. However, the deleterious impact of some kinds of NSAIDs on bone remodeling and fracture healing remains controversial [79]. Therapeutic doses of diclofenac, indomethacin or ketorolac would result in cell death during the osteoblast cultures; they may suppress bone formation and impair bone remodeling by causing cell cycle arrest in G0/G1 phase [80, 81]. In contrast, Arpornmaeklong et al. [82] found that indomethacin and celecoxib inhibit cell growth, but how alkaline phosphatase and osteocalcin synthesis were determined by them is unclear [76]. Studies of animal models indicated that celecoxib inhibits fracture consolidation at doses of 2–8 mg/kg/day, whereas other studies did not report any effects of celecoxib on fracture healing at doses of 1, 3, 10 and 50 mg/kg/day. Acetic acid and propionic acid [83] NSAIDs are known to play negative roles in bone healing; in contrast, naproxen, a propionic acid NSAID, inhibited osteoclastic activity and bone resorption and prevented transient loss of bone mass and structural deterioration. Numbers of research has reported that NSAIDs treatment can reduce bone generation to decrease the incidence of heterotopic bone formation following hip and femoral neck fractures [84]. NSAIDs are considered inhibitors of bone formation in experimental models of bone ingrowth into implants [85]. Although little is known about the mechanism underlying NSAID-induced reduction in bone formation, research in the latest decade continues to be in agreement with the results of earlier studies and strongly emphasizes the negative roles of traditional NSAIDs on bone healing [24]. Table 1 presents a list of animal studies regarding the functions of NSAIDs in bone remodeling and fracture healing. Several studies have shown that increased BMD indicates low bone-resorption rates in patients receiving NSAIDs, as measured by relevant biochemical markers [86]. The other study found modest beneficial effect on BMD, but no protective function on subsequent risk of fractures by taking NSAIDs 5–7 times/week [87]. However, another studies showed that NSAIDs have no effect on bone remodeling by regular and incidental use [88]. The contrasting findings may result from factors including treatment duration, dosage, and experimental species. For example, celecoxib, a COX-2 specific inhibitor, was found to impairing fracture healing in dose of 2 or 4 mg/kg/day [89]. The propionic acid NSAIDs, such as flurbiprofen, increased bone resorption [90] and aggravated the bone loss at the high dose of 1200 or 2400 mg/day [91, 92]. In contrast, Morton et al. [93] was unable to detect any effect of BMD in patients treated with acetic acid NSAIDs. Table 2 presents a review of clinical effects of aspirin and NSAIDs on BMD and fractures healing.

**Table 1:** Animal studies regarding the functions of NSAIDs in bone remodeling and fracture healing

Category	NSAIDs	Dosage	Model	Function	Refs.
COX-2 inhibitor	Celecoxib	3, 10, 50 mg/kg/day	Mice	No effect on fracture healing	[92, 93]
		10 mg/kg/day	Rat	No effect on fracture consolidation	[98]
		2, 3, 4, 6, 8 mg/kg/day	Rat	Inhibit fracture consolidation with all doses over time course	[95–98]
		1 mg/kg	Rat	No effect on fracture consolidation	[99]
Acetic acid	Diclofenac	1, 2 mg/kg/day	Rat	No effect on fracture healing	[100]
	Etodolac	20 mg/kg/day	Rat	Inhibits fracture consolidation	[101]
	Indomethacin	2 mg/kg/day	Rat	Inhibits bone remodeling	[101]
		Short-term	Rat	Inhibits fracture consolidation	[102]
Propionic acid	Ibuprofen	30 mg/kg/day	Mice	No effect on fracture healing	[103]
		30 mg/kg/day	Rat	No effect on fracture consolidation	[104]
		30 mg/kg/day	Rat	Inhibits fracture consolidation	[105]
		7.5, 17, 34 mg/kg/day	Rabbit	Inhibits fracture healing with dose response	[83]
	Naproxen	4–28 mg/L	Rat	Inhibit osteoclastic activity and bone resorption	[106]
		100 mg/L	Rat	Prevents transient loss of bone mass and structural deterioration	[107]
		10 mg/kg/day	Rat	No effect on BMD and biomechanical properties of spine and femur	[108]

*NSAIDs* nonsteroidal anti-inflammatory drugs, *BMD* bone mineral density

**Table 2:** Clinical effects of aspirin and NSAIDs on BMD and skeletal regeneration

Category	NSAIDs	Usage and dosage	Function (longest time point, month)	Refs.
NSAIDs	NSAIDs	Daily use	No effect on bone resorption	[23]
		Regular and incidental use	No effect on bone remodeling	[88]
		5–7 times/week	Modest beneficial effect on BMD, no protective effect on subsequent risk of fractures	[87]
COX-2 inhibitor	Celecoxib	200–400 mg/day	Reduces radiographic progression of structural damage of ankylosing spondylitis (24 m)	[109]
		200 mg/day	No effect on osteointegration of cementless total hip stems	[110]
		2 or 4 mg/kg/day	Impairs fracture healing	[89]
Acetic acid	Diclofenac	150 mg/day	Inhibits bone resorption	[111]
	Indomethacin	75 mg/day	No difference in fracture healing grade distribution	[112]
		100 mg/day	Impairs fracture healing grade	[101]
Propionic acid	Flurbiprofen	200 mg/day	Decreases excellent functional result	[113]
		2400 mg/day	Bone loss around implants (6 m)	[91, 92]
		1200 mg/day	Increases bone resorption	[90]
	Naproxen	1000 mg/day	Bone defect fill and resorption (9 m)	[114]
	Flurbiprofen	100 mg/day	Inhibits periosteal bone formation Inhibits bone resorption	[115]
Enolic acid	Piroxicam	20 mg/day	No effect on BMD and fracture healing	[116]

*NSAIDs* nonsteroidal anti-inflammatory drugs, *BMD* bone mineral density

## Discussion

Aspirin administration has controversial spatiotemporal effects in the skeletal system according to therapeutic doses applied and the clinical conditions of patients. Although several theories about the role of high-dose aspirin in inhibiting COX-2 and PGs in bone remodeling and destructive bone diseases has been reported, many basic questions remain unclear. For example, it is still unknown in which cells and when COX-2 is expressed during bone remodeling and fracture healing. Furthermore, the repertoire of PGs and other eicosanoids produced in bone remodeling is unknown, and it is unclear whether loss of activity in one of the arachidonic acid-metabolizing enzymes would affect the function of the remaining enzymes. Owing to involvement of inhibition in COX-1, COX-2, and PG pathways, the exact mechanisms of aspirin in bone remodeling are less conclusive. The COX- and PGs-independent pathways involved in the bone remodeling appear to either inhibit osteoclast differentiation or promote bone formation. Although we cannot overlook the negative effects of high-dose aspirin treatment on BMD and bone regeneration in human studies, there is no conclusive evidence to deny patients the analgesic benefits of these drugs for managing fractures. Owing to its anti-platelet effect, which prevents heart attacks and strokes, aspirin at a dose of 75 or 81 mg/day is commonly used in elderly patients requiring long-term treatment. In vitro studies and animal studies have indicated that low-dose aspirin could increase osteoblast formation and decrease osteoclast differentiation, thereby increasing mineralized tissue formation [26].

The clinical effects of regular use of aspirin on BMD and skeletal regeneration in elderly patients remain conflicting conclusions of previous epidemiological studies [26]. Bauer et al. found that daily aspirin use (5–7 days/week, at least 1 year) in white women aged > 65 years was associated with an increase in axial BMD as compared to non-users. However, this increase in BMD was not accompanied with a clinical protective effect on the fracture risk [87]. In another study including African–American ethnicity and men, current users of aspirin had higher BMD of the total body than nonusers, but data on fracture risk were not presented [11]. However, these studies did not demonstrate the differences and similarities in the effects of different doses of aspirin on BMD and fracture risk. High-dose aspirin is effective for patients with similar conditions such as degenerative bone and joint diseases and for those taking many medications. Vestergaard et al. [94] reported that the overall fracture risk is slightly reduced in OA patients with recent use of high-dose aspirin (≥ 500 mg/day) for the treatment of pain and inflammation. On the other hand, low-dose aspirin is commonly used for prevention of thromboembolic events in elderly people. Vestergaard et al. [41] found that the use of low-dose aspirin increased risks of overall fracture risk and hip fracture, although the effect size was only marginal. A recent study of Bonten et al. [95] demonstrated that the regular use of low-dose aspirin is not associated with lower BMD in general population. These findings between aspirin use and increased fracture risk is in contrast to the previous in vitro and in vivo findings. The disease itself and the medications used to treat it might confound the results of epidemiological studies on aspirin use. Furthermore, there are no conclusive criteria to define the precise doses of aspirin for in vitro investigations that correspond to the doses in human or animal studies. Therefore, it is difficult to elucidate the roles of aspirin in human studies. Anna et al. initiated a well-designed randomized controlled trial to assess whether daily using low-dose aspirin can benefit for bone health and prevent the fractures in elderly people [96]. Their research conclusions are expected to be a strong evidence for clinical usage of low-dose aspirin for skeletal regeneration.

NSAIDs are involved in bone remodeling and affect fracture healing [97], however, there is no clinically relevant result to prove the association between NSAIDs use and bone destructive diseases. Although all NSAIDs share a common mechanism of action, they vary widely in their clinical outcome because of their physicochemical differences [78]. This disparity could be due to several reasons. First, the differential effects of COX inhibition by different doses of NSAIDs on bone are not completely understood. Second, epidemiologic studies on the use of aspirin or NSAIDs may be confounded by age, sex, obesity and ethnicity, and involve inconformity of BMD, which may be a source of bias. Finally, patient compliance to the duration and frequency of taking medication should be considered.

## Conclusions and perspective

In summary, low-dose aspirin might be a promising drug for relieving bone diseases that are related to abnormal bone remodeling, such as osteoporosis. Additionally, it is considered a suitable thrombosis phylactic agents following orthopedic operations. In contrast, the role of high-dose aspirin in bone remodeling is difficult to elucidate owing to its dual effects on osteoclast activity and osteoblast formation. Other NSAIDs have similar problems as high-dose aspirin. However, according to the promotional functions of COX-2-dependent PGs in angiogenesis [64, 65], COX-2 inhibitors and high-dose aspirin might be associated with a delay or nonunion of fracture healing, although the evidences for this finding are conflicting. Therefore, the clinical application of high-dose aspirin and NSAIDs for relieving pain in fractures should be more carefully and strictly controlled, particularly in the early stage of angiogenesis and bone repair. Low-dose aspirin used for the prevention of ACS might benefit bone health by protecting against the destruction of bone tissues and decreasing the risk of fractures. Further investigations are needed to fully illuminate the potential modulatory roles of aspirin in bone remodeling, especially with different doses, and determine the functions of aspirin and NSAIDs in relation with BMD and orthopedic operations.

## Data Availability

Not applicable.

## Abbreviations

NSAIDs: non-steroidal anti-inflammatory drugs
HSCs: hematopoietic stem cells
MSC: mesenchymal stem cell
RANKL: receptor activator of nuclear factor-κB ligand
BMD: bone mineral density
COX-1: cyclooxygenase-1
COX-2: cyclooxygenase-2
PGE2: prostaglandin E2
TXA2: thromboxane A2
ACS: acute coronary syndromes
TRAP: tartrate-resistant acid phosphatase
OVX: ovariectomized
OPG: osteoprotegerin
IFN-γ: interferon-γ
TNF-α: tumor necrosis factor-α
TGF-β1: transforming growth factor β-1
NO: nitric oxide
BMP-2: bone morphogenetic protein 2
FGF-2: fibroblast growth factor 2
5-LO: 5-lipoxygenase
RANK: receptor activator of nuclear factor-κB
PKG: protein kinase G
PKA: protein kinase A
AMPK: adenosine monophosphate-activated protein kinase
